# The Paradox of a Phagosomal Lifestyle: How Innate Host Cell-*Leishmania amazonensis* Interactions Lead to a Progressive Chronic Disease

**DOI:** 10.3389/fimmu.2021.728848

**Published:** 2021-09-07

**Authors:** Matheus B. Carneiro, Nathan C. Peters

**Affiliations:** Snyder Institute for Chronic Diseases, Departments of Microbiology, Immunology and Infectious Diseases, Cumming School of Medicine and Comparative Biology and Experimental Medicine, Faculty of Veterinary Medicine, University of Calgary, Calgary, AB, Canada

**Keywords:** *Leishmania*, *Leishmania amazonensis*, neutrophils, monocytes, macrophages, dendritic cells, phagolysosome

## Abstract

Intracellular phagosomal pathogens represent a formidable challenge for innate immune cells, as, paradoxically, these phagocytic cells can act as both host cells that support pathogen replication and, when properly activated, are the critical cells that mediate pathogen elimination. Infection by parasites of the *Leishmania* genus provides an excellent model organism to investigate this complex host-pathogen interaction. In this review we focus on the dynamics of *Leishmania amazonensis* infection and the host innate immune response, including the impact of the adaptive immune response on phagocytic host cell recruitment and activation. *L. amazonensis* infection represents an important public health problem in South America where, distinct from other *Leishmania* parasites, it has been associated with all three clinical forms of leishmaniasis in humans: cutaneous, muco-cutaneous and visceral. Experimental observations demonstrate that most experimental mouse strains are susceptible to *L. amazonensis* infection, including the C57BL/6 mouse, which is resistant to other species such as *Leishmania major*, *Leishmania braziliensis* and *Leishmania infantum*. In general, the CD4^+^ T helper (Th)1/Th2 paradigm does not sufficiently explain the progressive chronic disease established by *L. amazonensis*, as strong cell-mediated Th1 immunity, or a lack of Th2 immunity, does not provide protection as would be predicted. Recent findings in which the balance between Th1/Th2 immunity was found to influence permissive host cell availability *via* recruitment of inflammatory monocytes has also added to the complexity of the Th1/Th2 paradigm. In this review we discuss the roles played by innate cells starting from parasite recognition through to priming of the adaptive immune response. We highlight the relative importance of neutrophils, monocytes, dendritic cells and resident macrophages for the establishment and progressive nature of disease following *L. amazonensis* infection.

## Introduction

Phagocytes and phagocytosis were first described by Elie Metchnikoff in 1883. At that time phagocytosis was primarily described in frogs and associated with homeostasis, nutrition and tissue reabsorption. Later, Metchnikoff described how this process could also act as a protective mechanism against pathogens (1). “Infection, a struggle between two organisms”, was the title Metchnikoff gave to the first of a series of lectures he delivered in 1891 (2), a title that accurately described the complex relationship, from an evolutionary perspective, between the host immune system and infectious pathogens. After 130 years, the challenge to understand how best to treat infectious diseases caused by pathogens that employ phagocytes as hosts for infection, replication and persistence *via* prophylactic vaccination, immunotherapy, or antibiotics remains. In the case of the intracellular parasite *Leishmania*, the infection poses significant challenges to treatment and prevention due to multiple mechanisms of immune evasion that allow the parasite to infect the very cells that the immune system employs to eliminate them (3, 4). Since the late 1970s *Leishmania* parasites have been described as phagosomal pathogens that reside and proliferate within phagocytic cells (5–8). We now understand that even a single *Leishmania* parasite transmitted by a sand fly bite is sufficient to establish infection (9, 10), corroborating data discussed throughout this review that innate mechanisms of immunity are not sufficient to provide protection and that the development of an adaptive immune response, mediated largely by Th1 CD4 cells, is required to activate phagocytic cells (11).

Leishmaniasis is a vector-borne disease associated with different clinical manifestations, determined by the intersection of the parasite species and the host immune response (12). In this review, we will discuss the paradoxical relationship between *L. amazonensis* (*L.a.*) parasites and phagocytic cells during cutaneous Leishmaniasis. Infection with *L.a.* is intriguing as it has been associated with a remarkably diverse clinical manifestations including localized cutaneous leishmaniasis (LCL), borderline disseminated cutaneous leishmaniasis (BDCL), anergic diffuse cutaneous leishmaniasis (ADCL) and, less frequently, with mucosal and visceral leishmaniasis (13, 14). We will focus on how living in a phagosome represents a challenge to *L.a.* and how this parasite has evolved to survive not only against innate mechanisms of immunity, but also to take advantage of Th1 immunity for the establishment and perpetuation of disease.

## No Place Like Home: The Biology of the *Leishmania* Phagolysosome

### Phagocytosis

**Figure 1:**
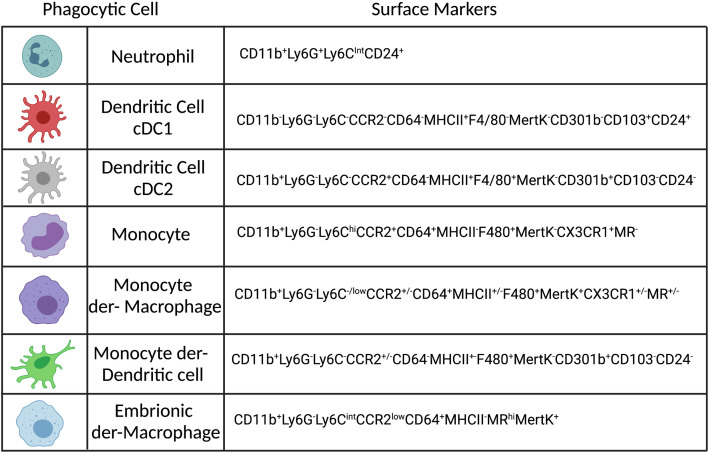
Phenotypes of the most abundant phagocytic cells in the dermis of *Leishmania* infected mice. Created with BioRender.com.

Phagocytosis is an important biological function mediated by monocytes, macrophages, neutrophils and dendritic cells (DCs), see **Figure 1**, that plays a role in both inflammation and homeostasis, where it can mediate both pathogen elimination and tissue healing (15). Phagocytosis is a complex and well-regulated process that has been reviewed in detail elsewhere (16). Briefly, phagocytosis is initiated by the binding of specific molecules expressed by pathogens, or the host, to surface receptors expressed on phagocytic cells and is therefore a contact-dependent process. The phagocyte receptors associated with *Leishmania* phagocytosis are either opsonic, when host-particles (opsonins) bind to the pathogen or non-opsonic, when they bind directly to pathogen-associated molecular patterns (PAMPs). Opsonic receptors are: FcγRs (CD64, CD32, CD16), complement receptors (CR1-4, Mac-1) and fibronectin receptors (a5B1), which bind respectively to antibodies (abs), complement and fibronectin. Examples of non-opsonic receptors are mannose receptor (MR or CD206), Dectin-1 and DC-SIGN (17–21). Specific receptors can also recognize apoptotic cells, such as TIM-1, TIM-4, which recognized phosphatidyl serine (PS), exposed on the membrane of dying cells, or MertK (tyrosine-protein kinase Mer), which binds to GAS6 (growth arrest-specific 6), a molecule that bridges PS to the membrane (22). In addition, *in vitro*, *L.a.* has been shown to use Toll-like receptor (TLR) 2 to infect neutrophils (23) and can also subvert endocytosis during cell plasma membrane repair to invade non-phagocytic cells, such as fibroblasts (24). As reviewed by Ueno and Wilson, the receptor involved in parasite entry may influence parasite fate, as it can influence phagolysosome maturation, reactive oxygen species (ROS) production and phagocytic cell activation (25). After binding to the phagocytic receptors, pseudopods surround *Leishmania* parasites and form the phagosome. During phagocytosis of *L.a.* metacyclic promastigotes, entrance is often mediated *via* the cell body rather than the flagellum, in a process that can take up to 10 minutes *in vitro* employing bone-marrow derived macrophages (BMDMs) (26). The engulfment of either promastigotes or amastigotes is associated with an arrangement of the cytoskeleton and a transient polymerization of F-actin around the parasites (26, 27).

### Formation and Maturation of the Parasitophorous Vacuole

This initial phagosome will undergo a maturation process mediated by fusion with endosomes and lysosomes. After internalization, the early phagosome, characterized by moderate pH acidification, will fuse with early endosomes, a process that relies on the expression of Rab5, a GTPase (guanosine triphosphatase), that mediates membrane fusion events (16). The late phagosomes are formed when early phagosomes fuse with late endosomes. In this stage there is a shift from Rab5 to Rab7 at the phagosome membrane and the lumen becomes more acidic, due to an increase in the expression of V-ATPases (which translocate protons H^+^ from the cytosol to the phagosome at the cost of an ATP). The Rab7 mediated fusion of the late phagosome with lysosomes characterizing the formation of the phagolysosome or parasitophorous vacuole (PVs). The phagolysosome is characterized by membrane expression of lysosomal markers such as lysosomal associated membrane protein 1 and 2 (LAMP-1 and LAMP-2) and the presence of cathepsins, proteases and lysozymes in the lumen. In addition, *L.a.* PVs are considered hybrid compartments as the phagosome also presents molecules associated with the endoplasmic reticulum (ER) pathways such as calnexin (28).

#### *Leishmania* Survival in the Phagolysosome

Phagolysosomes are highly acidic, oxidative, contain antimicrobial peptides, proteases and restrict nutrient access, representing a highly hostile environment for an intracellular pathogen (16, 29). However, many intracellular pathogens have evolved different mechanisms to either avoid phagocytosis, escape from the phagolysosome or to resist phagolysosomal effector mechanisms (29). In contrast, *Leishmania* parasites are adapted to survive and replicate within phagolysosomes and different species of *Leishmania* have developed different strategies to do so. Survival primarily appears to be facilitated by allowing time for the parasite to differentiate into an amastigote before the hostile mature phagolysosome is formed. For instance, during *in vitro* infection of macrophages, promastigotes of *L. donovani* and *L. major (L.m.)* can delay phagolysosome maturation by preventing acidification (30), phagosome-endosome fusion (31) or NADPH (nicotinamide adenine dinucleotide phosphate) oxidase assembly (32). Curiously, *L.a.* parasites do not seem to interfere with phagolysosome maturation, an observation that is somewhat counterintuitive. Rather, several studies show that acidification, fusion between the phagosome and endosomes/lysosomes, lysosomal enzymes and protease activities and the expression of lysosome markers (Rab7, LAMP1, V-ATPase) are all present on *L.a.* containing phagolysosomes (26, 33–37). In fact, the formation of the *L.a.* phagolysosome is relatively rapid, occurring within 30 minutes post infection, when the phagolysosomes of most infected macrophages have already acquired lysosomal features in the lumen and membrane. This process occurs faster when the infection is initiated by the amastigote form of the parasite but is otherwise quite similar compared to promastigotes and it is not influenced by previous exposure to IFN-γ activation (26). Altogether, *Leishmania* amastigotes are extremely well-adapted to survive and proliferate inside mature phagolysosomes and *L.a.*, in particular, does this without significantly interfering with this host defense mechanism compared with other species.

The structure of phagolysosomes differs when containing different *Leishmania* species. During *L. major* and *L. donovani* infection, the PVs are tight and harbor a single amastigote, while infections with species from the *L. mexicana* complex, such as *L.a.*, generate large communal PVs that contain several amastigotes attached to the vacuole membrane (36). It has been suggested that the increased size of *L.a.*-containing vacuoles would dilute the effect of antimicrobial molecules, compromising parasite killing and favoring growth (38, 39), which could explain why killing *L.a.* amastigotes is harder to achieve then other species such as *L.m *(40, 41). The development of large PVs depends on the accumulation of CD36 at the sites where amastigotes attach to the phagosome membrane, suggesting that this process could occur either during or after amastigote differentiation. CD36^-/-^ BMDMs infected with *L.a.* developed tight PVs that restricted *L.a.* growth but had no impact on *L.m.* growth (39). *In vitro*, the PV enlargement process is rapid and seen 8 h post-infection with promastigotes, reaching their maximum volume by 24 h (26, 39). CD36 likely induces PV enlargement by enhancing the fusion with late endocytic vesicles, more specifically with lysosomes (39). As fusion with ER vesicles has also been shown to participate discretely in the enlargement of *L.a.* PVs (42), it is possible that CD36 enhance this fusion pathway as well. Likely as a mechanism of defense, *in vitro* infected macrophages respond to *L.a.* infection by enhancing the lysosomal trafficking regulator (LYST)/Beige expression, a protein complex that limits the size of lysosomes, therefore reducing the size of the PVs and restricting parasite replication (38). However, it is unlikely that during *in vivo* infection the levels of LYST/Beige expression by host infected cells is enough to impact parasite growth, as chronic disease is associated with large PVs containing several amastigotes within mononuclear cells (43, 44). In addition, the engagement of CD36 driving PV enlargement is not associated with changes on LYST/Beige expression (39). Importantly, while the PV size is associated with *L.a.* growth restriction in tight PVs (39, 45), it did not impact parasite killing mediated by IFN-γ+ LPS on *in vitro* activated BMDM, arguing against the idea that large PVs would dilute microbicidal molecules, providing a shield to the parasites. However, direct *in-vitro* activation by IFN-γ + LPS might induce a stronger activation phenotype on host cells compared to *in vivo* infection and therefore it remains a possibility that the large PVs in *L.a.* infection provide a niche in host phagocytic cells that don’t get sufficiently activated.

Differences in the properties of phagolysosomes have also been reported between different phagocytic cells and this is relevant to *Leishmania* infection. Neutrophils are known for being able to produce up to 20 times more ROS than macrophages, which in combination with a lower expression of V-ATPase, due to the high activity of NADPH oxidase, characterize an early alkaline environment in the PVs (46–48). In addition, due to a lack of the endosomal pathway in neutrophils, the phagosome mostly fuses with the preformed granules found in these cells, which also contain NADPH oxidase in their membrane, contributing to higher ROS production (47). Fusion of phagosomes with azurophilic granules containing myeloperoxidase (MPO) has been described during infection of human neutrophils *in vitro* with *L. major* and *L. donovani* (49), however, little is known about the phagolysosome biology of neutrophils during *Leishmania* infection and an important question that remains to be elucidated is if the differences in phagolysosome maturation influences promastigote-amastigote differentiation in different phagocytic cells and how this might impact the development of disease.

#### Nutrient Acquisition in the Phagolysosome

The life experience of phagolysosome exposed amastigotes, including changes in temperature, pH, O_2_, and restricted access to nutrients, is significantly different compared to promastigotes, which are largely restricted to the sand fly vector midgut. In fact, some of these changes have been associated with the differentiation process from promastigotes to amastigotes, including the requirement for higher temperature and lower pH for the propagation of axenic amastigotes *in-vitro* (50). Interestingly, for *L.a.*, ROS production and the acquisition of iron play critical roles in this process. The phagolysosome restricts the iron access to the parasites. Iron is a key cofactor to many enzymes and regulates important physiological pathways in different organisms. Iron deprivation induces an up-regulation of *Leishmania* iron transporter 1 (LIT1), that maintains internal iron content while reducing parasite proliferation. Parasites lacking LIT1 continue to proliferate until they show a robust cell death rate (51). In addition, *Leishmania* require iron as a co-factor for the activity of iron superoxide dismutase (FeSOD), a key enzyme that converts superoxide, mostly derived from parasite mitochondria, into hydrogen peroxide (H_2_O_2_), which drives amastigote differentiation (51, 52). In addition to iron, amastigotes need to uptake other nutrients from the phagolysosome lumen or cytoplasm including several essential amino acids, lipids, vitamins and purines, which are essential for parasite survival and growth (53). This uptake relies on phagosome-endosome fusion, on transporters present at the PVs membrane and on transporters expressed by the parasites themselves (53–56). A critical auxotrophic micronutrient for amastigotes is L-arginine. This amino acid is the only source for polyamines production, essential for parasite survival (57). *L. amazonensis* express their own arginase enzyme that hydrolyses L-arginine into L-ornithine and urea. Then ornithine decarboxylase (ODC), catalyzes the enzymatic decarboxylation of L-ornithine into polyamines (58). Amastigotes can also access polyamines generated by host cytoplasmatic arginase -1 (Arg1) activity (59). During infection, host cells increase the consumption of L-arginine as this amino acid is required for the activity of both arginase and inducible nitric oxide synthase (iNOS) enzymes. The up regulation of the receptor cationic amino acid transporter 2b (CAT-2B), modulated by IFN-γ, allows the necessary upregulation of L-arginine uptake. Interestingly, *Leishmania* parasites can sense the availability of L-arginine in their environment and change the expression of their own amino acid transporters (60–64).

### Induction of Respiratory Burst and Nitric Oxide Production

Pathogen engulfment triggers the assembly and activation of the enzyme complex NADPH oxidase in phagocytic cells. This enzyme reduces O_2_ into superoxide that can mediate damage to the parasite (47). Both *in vitro* infection of macrophages and neutrophils with either *L.a.* promastigotes or amastigotes induces this respiratory burst process (65, 66). However, the absence of a functional NADPH oxidase in mice lacking the membrane catalytic unit gp91 (Nox2) does not impact parasite survival significantly to have an impact on the establishment of disease or parasite loads over the course of infection (65, 67, 68). The production of nitric oxide (NO) requires the activity of iNOS, whose expression is induced by TNF, IFN-γ, IL-1β, and TLR ligands such as LPS (43, 69–71). L-arginine is the substrate for iNOS, therefore robust uptake of this amino acid is required when phagocytic cells are activated to ensure sustained and robust NO production (47, 72). iNOS expression can be found at the phagosome but its primary expressed on other vesicles in the cytosol (73, 74). As NO is a relatively stable uncharged molecule it can cross membranes such as the PV and the plasma membrane (75). NO mediates its cytotoxic effect by: a) reacting with heme-cytochrome C oxidase in pathogen mitochondria to initiate ROS production; b) compromising DNA synthesis by inhibition of ribonucleotide reductase activity; c) causing direct DNA damage (47, 76); d) reacting with ROS forming peroxynitrite anion (ONOO-), which is a stronger oxidant that promotes pathogen killing by compromising mitochondrial respiration and mediating tyrosine nitration that impacts proteins structure and function (77). Finally, NO can inhibit overall metabolism activity in *Leishmania* parasites restricting their growth (78). While ROS production does not provide protection against *L.a*, absence of NO is associated with enhanced parasite growth and pathology (44, 71, 79). *L.a.* can interfere with iNOS/NO production in different ways: by expression of ectonucleotidases, such as ecto-nucleoside triphosphate diphosphohydrolase (E-NTPDase), an enzyme that provides AMP for adenosine production, which then binds to adenosine A2B receptors on macrophages decreasing NO production and inhibiting TNF and IL-12 production (80); by activating NF-κB repressors (81, 82) or by inhibiting iNOS activity (83).

## The Initial Events: *Leishmania*-Phagocyte Interactions

### Parasite Antigens and Innate Recognition

Innate cells detect pathogens, including *Leishmania* parasites, by recognizing PAMPs through the expression of pattern recognition receptors (PRRs). PRRs are expressed on the cell surface, in the cytoplasm, or are secreted into the blood stream or tissue fluids (84).

*Leishmania* metacyclic promastigotes are the infective stage of the parasite predominantly transmitted into the dermis of mammalian hosts by infected sandflies during acquisition of a blood meal (10, 85, 86). Metacyclic promastigotes are characterized by the presence of a thick glycocalyx on the cell surface where different proteins are expressed, representing a source for PAMPs. The most common components expressed on promastigotes are glycophosphatidylinositol lipids (GIPLs), lipophosphoglycan (LPG), proteophosphoglycan (PPG) and the GP63 zync-metalloprotease. Most of these molecules are attached to the parasite surface by glycosylphosphatidylinositol (GPIs) anchors (87). The amount of their expression can vary between the different parasite life-cycle stages and the structure of these components is different between different species of *Leishmania*, thereby explaining variations in the role played by these glycoconjugates following infection with different species (88).

Because of the pool feeding feature of sand flies, the first innate immune mechanism that challenges *Leishmania* survival is the exposure to complement components in the serum (89). *L.a.* metacyclic promastigotes are resistant to complement through at least two different mechanisms: cleavage of C3b into iC3b by GP63 or by interfering with correct C9 attachment to the parasite surface, in both cases avoiding the formation of the membrane attack complex (MAC), enabling parasite survival (90–92). After evading complement, *L.a.* must be phagocytosed by phagocytic cells to establish a niche for survival/replication. Interestingly, the presence of iC3b on the parasite surface is not only important to escape lysis mediated by the MAC, but also facilitates parasite phagocytosis by CR1/CD11b (93). It is possible that receptors such as CR3 bind simultaneously to both opsonin (iC3b) and the promastigote (LPG) in different binding sites enhancing phagocytosis (94). In addition, GP63 secretion can digest collagen type IV and fibronectin in an *in vitro* extracellular matrix, which might facilitate parasite-phagocyte encounters in the skin (95).

#### The Role of Pattern Recognition Receptors During *Leishmania* Infection

When phagocytic cells bind to and internalize *Leishmania* parasites host PRRs can be activated by PAMPs. The impact of TLR activation on leishmaniasis depends on parasite species, host genetic background, cell subset and has been linked to both anti and pro-inflammatory responses (96). Purified *L.a.*-LPG activates TLR2, triggering the production of type I interferon leading to activation of the IL27/IL-10 axis and inducing superoxide dismutase (SOD) expression, which impairs macrophage activation and favors parasite replication both *in vitro* and in murine infections (97–101). The production of type I interferon and IL-27/IL-10 axis is mediated by activation of ds-RNA dependent kinase (PKR), a PRR, which in this scenario is activated by type I interferon and IL-27 (99, 100). Importantly, in a model of co-infection of *L.a.* with an Amazonian Phlebovirus, which can infect sandflies, an exacerbation of lesion size and parasite load also correlated with an increase on IFN-β/IL-10 production associated with PKR activation (102). In addition, TLR2-deficient DCs infected *in vitro* showed higher IL-12p40 levels and induced stronger activation of naïve CD4 T cells as measured by higher replication rate and IFN-γ production compared to Wt DCs (103). Lastly, skin biopsies from patients infected with *L.a.* showed a higher expression of IFN-β/PKR for the more severe form of the disease, anergic diffuse cutaneous leishmaniasis (ADCL) compared to localized cutaneous leishmaniasis (LCL) (99). Thus, the TLR2/Interferon type I/PKR pathway plays a significant role in parasite evasion. Despite the pathogenic role played by TLR2 during *L.a.* infection, Myd88 deficiency is associated with higher susceptibility to disease, thus suggesting additional roles mediated by different TLRs that also signal *via* Myd88 (71, 104). In this context, TLR4, which can also be activated by LPG, is associated with both NO and TNF production following *in-vitro* macrophage infection (104, 105). The role played by TLR9 is controversial, *L.a.* amastigotes can release DNA on micro-vesicles, activating endosomal TLR9, which required both Myd88 and TRIF to induce CD200, which blocks iNOS expression, resulting in a transient higher resistance in TLR9^-/-^ mice compared to Wt mice, in early but not chronic phases of the disease (106, 107). Lastly, the expression of TLR2, TLR4 and TLR9 is different during different clinical manifestations of *L.a.* infection in humans. Specifically, LCL showed a higher density of TLR2 expression compared to TLR4 and TLR9 by CD68^+^ macrophages from skin biopsies. On the other hand, for ADCL and BDCL forms, the density of TLR9 expression is higher comparing to TLR2 and TLR4. Importantly, no correlation between the expression pattern of TLRs and production of TNF, IL-10 or TGF-β by T cells were found (108).

Another important family of PRRs are the NOD-like receptors (NLRs), which work as a sensor for intracellular molecules. NLRs are important components in the inflammasome complex. In general, activation of the inflammasome multiprotein complex in the cytosol leads to activation of caspase-1 that cleaves the inactive proinflammatory cytokines (pro-IL-1β and pro-IL-18) into their respective active forms (109). The activation of the inflammasome through NRLP3 depends on ATP, released by dying cells at the site of infection, and ROS production by NADPH oxidase, released during parasite phagocytosis. In addition, *L.a.* LPG triggers a non-canonical pathway of inflammasome activation mediated by caspase-11, although it is not yet known how LPG from the parasites gains access to the host cell cytoplasm to activate the inflammasome complex (110–112). In general, IL-1β production during *Leishmania* infection has been associated with different disease outcomes: pathology in the case of *L. major* and *L. braziliensis* infection, dissemination following *L. infantum* infection, and a protective role against *L.a *(71, 113–115). BMDMs, previously primed with LPS, and infected by *L.a.* promastigotes induce IL-1β production, mediated by inflammasome activation. IL-1β was then shown to induce iNOS production, having a long-term impact on disease, as assessed by higher susceptibility of mice deficient for NLRP3, CASP1 and CASP11 (71, 110). IL-18 production, on the other hand, is associated with higher pathology and parasite loads during *L.a.* infection by an unknown mechanism (116). Interestingly, activation of the inflammasome seems to depend on the parasite stage. Different from promastigotes, infection by amastigote forms of *L.a.* inhibit several components of inflammasome activation including NRLP3, NLRP4 and AIM2 by modulating epigenetic changes at H3 (hypoacetylation) in macrophages (117). In addition, infection of DCs *in vitro* with *L.a.* amastigotes also inhibited inflammasome activation resulting in less IL-1β and IL-18 production (118). Considering the differences between how promastigotes and amastigotes modulate inflammasome activation it is not clear how inflammasome activation by promastigotes during the early stages of the infection would impact long-term disease by inducing iNOS expression, especially in the context of subsequent Th1-mediated iNOS production in response to IFN-γ. Currently, it is not known if inflammasome activation has an impact on infiltrating phagocytic cells during *L.a.* infection, thereby influencing permissive host cell availability. Another outstanding question is the impact of inflammasome activation on neutrophils, which represent important host cells infected by promastigote forms of the parasite, predominantly at acute stages of infection (10).

### Neutrophil-*Leishmania* Interactions in the Skin

Neutrophils are generated in the bone marrow in adult mammals and represent the most abundant leukocyte population in the bloodstream, allowing for rapid recruitment into damaged tissue sites. Neutrophil recruitment to damaged tissues, following sterile or infection-associated injury, is a hallmark of early innate immunity (119). Neutrophil migration from bone-marrow to the circulation relies on the balance between retention/mobilizing signaling mediated by the expression of CXCR4/CXCR2, respectively (120). Blood neutrophils are short-lived (half-life of around 10 h), which can increase up to 24 h following tissue infiltration under homeostasis, or even up to 7 days in the context of inflammation (119). Recently, by employing both parabiosis and a tamoxifen-inducible Cre recombinase at the *ly6g* locus, allowing for tracking neutrophil fates in different tissues, it was observed that neutrophil half-life depends on the tissue, and, interestingly, the skin, amongst the tissues analyzed, is the organ where neutrophils have the longest half-life (around 18 h) (121). In addition, transcriptome analyses from skin neutrophil suggest that these cells might regulate epithelial growth in the skin under steady state (121). During systemic infections, a 10x increase in neutrophil production can be observed, a process called emergency granulopoiesis, which can be triggered by the increase in serum levels of the growth factors granulocyte and macrophage colony-stimulating factors (G-CSF/M-CSF, respectively) and cytokines (IL-6, type I interferons), which regulate hematopoietic stem cells (HSCs) in the bone-marrow (122). Neutrophil recruitment is also enhanced during inflammation. Neutrophil chemotaxis is mediated by different receptors: I) chemokine receptors such as CXCR2, CXCR4 and CXCR1 (123). The inflammatory chemokine receptors CCR1, CCR2, CCR3 and CCR5, can also be expressed by neutrophils but do not play a role on neutrophil recruitment during either homeostasis or inflammation, and instead, appear to be more relevant for monocyte and eosinophil recruitment (124); II) lipid mediator receptor, such as leukotriene B4 receptor (LTB4R), which binds to leukotriene B4 and III) complement receptors, as C5aR, CR3 and C3aR (123). The acute neutrophil recruitment to wounded skin can also be impaired in the absence of a microbiome (125).

#### Neutrophil Behavior at the Dermal Site of *Leishmania* Deposition

**Figure 2:**
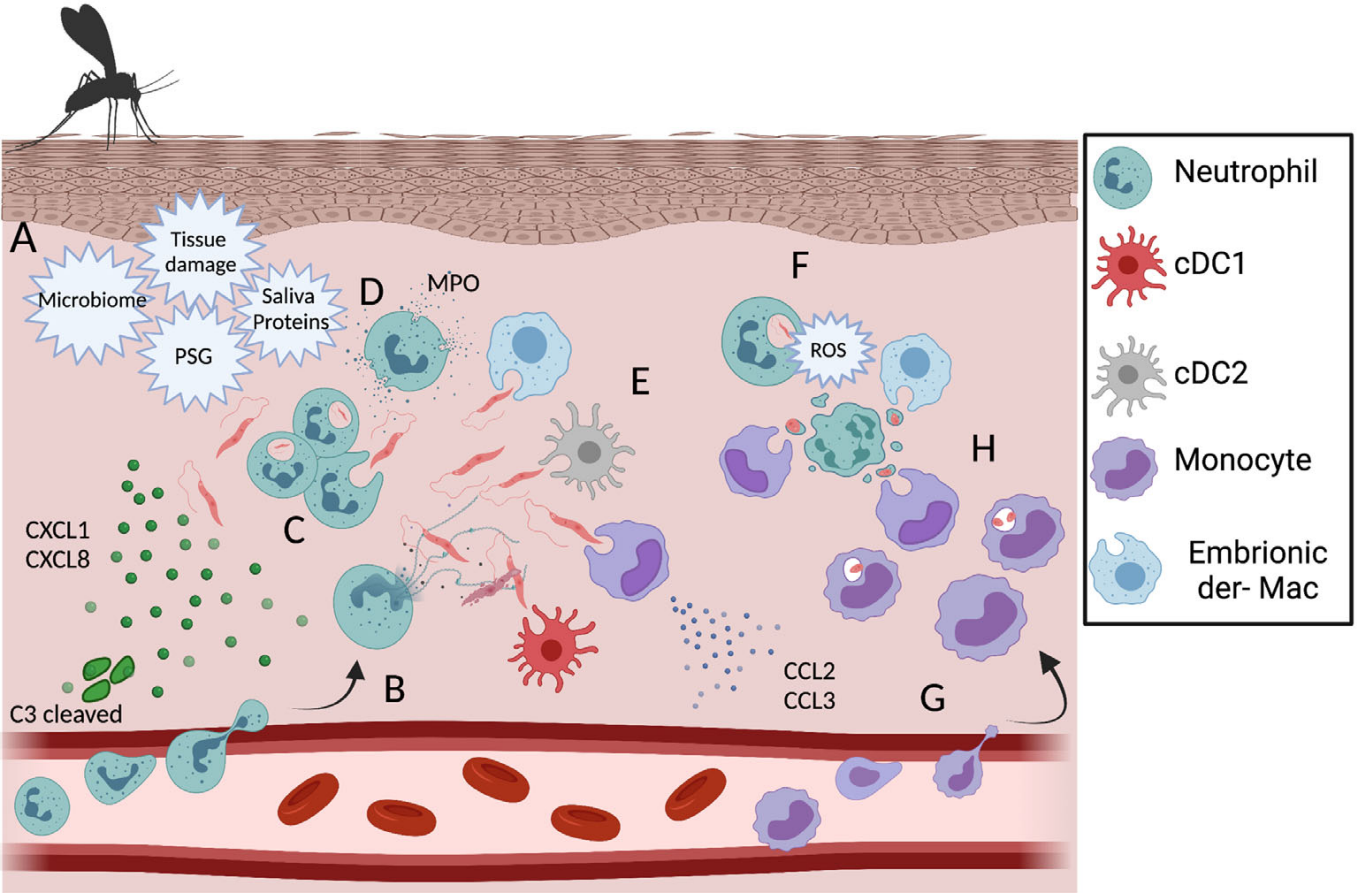
Dynamic of phagocytic cells during the acute phase of the immune response against *L. amazonensis* infection. **(A)** Recruitment of neutrophils is triggered by tissue damage, sand fly associated factors, chemokine production by keratinocytes and C3 cleavage. After recruitment to the dermis neutrophils can release NETs **(B)**, phagocytose **(C)** and degranulate **(D)** in response to *L.a.* infection. Other infected phagocytic cells during early infection include embryonic dermal macrophages, resident DCs and monocytes **(E)**. In response to metacyclic promastigote phagocytosis, neutrophils undergo respiratory burst and ROS mediated apoptosis **(F)**. In response to changes in the innate chemokine production profile, a wave of inflammatory monocytes is recruited to the dermis skin **(G)** and become infected by phagocyting infected neutrophils, apoptotic bodies, a process known as the “Trojan Horse” model of infection **(H)** or parasites freed by apoptotic neutrophils. Created with BioRender.com.

The employment of multiphoton intravital microscopy to image leukocyte migration into different tissues has made a great impact on the understanding of neutrophil behavior after transmigration, for instance, into wounded skin. Tissue damage following sand fly bite or needle inoculation is enough to induce a robust neutrophil infiltrate to the dermis in the minutes to hours post-infection with *L.m* (10, 126, 127). Needle inoculation of *L.a.* parasites also induce early neutrophil recruitment to the skin (128, 129), see **Figure 2**. In addition to tissue damage, neutrophil recruitment is also associated with vector associated components, such as the sand fly gut microbiota, sand fly saliva, and promastigote secretory gel (PSG), rich in proteophosphoglycans and secreted by the parasites in the vector midgut, as well as factors released by the host immune response to the bite, including CXCL1, CXCL2 and C3 cleavage (114, 130–132). Importantly, while the presence of *Leishmania* is not required to induce neutrophil recruitment, the presence of parasites or sand fly derived factors is likely required for persistent neutrophil swarming and maintenance of the ‘neutrophil plug’ that forms where the sand fly proboscis has penetrated through the skin. In contrast, sterile wounds are associated with a mostly transient presence of neutrophils (10, 126, 133). All of these factors are likely to contribute to the intense neutrophil cluster formation at sites of sand fly bite and, up to 24 h post infection, neutrophils represent a majority of the inflammatory host cells for most *Leishmania* species following transmission by sand fly bite or needle inoculation (10, 43, 134). Recently, sand fly transmitted parasites of a non-healing *L. major*-RYN strain that were distributed to dermal sites distal to the site of vascular damage and neutrophil cluster formation resulting from sand fly probing were shown to be phagocytosed by a more diverse population of phagocytes, including resident dermal macrophages (135), suggesting the localization of parasite deposition can influence host cell phenotype. The biological relevance of neutrophil swarming and cluster formation during the early phase of *Leishmania* infection may be to restrict parasite dissemination, as neutrophils tend to concentrate at sites of tissue damage where *Leishmania* are also found and may limit their subsequent spread following phagocytosis even if they are unable to kill the parasite. Second, the co-localization between neutrophil clusters and parasites might restrict tissue pathology induced by neutrophil-derived inflammatory factors to the wound associated with sand fly bite versus a more generalized distribution in the skin. In fact, the robust acute neutrophil recruitment after either sand fly or needle injection does not correlate with the persistent tissue pathology associated with cutaneous disease, as clinically relevant lesions typically only appear after the induction of adaptive immunity (10, 43, 128, 134).

#### Impact of Neutrophil Death on *Leishmania* Infection

In addition to sand fly bite associated factors, neutrophil cell death can also initiate or amplify neutrophil swarming and cluster formation (133). This observation is important in the context of *Leishmania* infection as parasite phagocytosis has been associated with the induction of neutrophil death. How fast *Leishmania* parasites induce neutrophil cell-death depends on parasite species and experimental settings (*in vitro versus in vivo*; and the source of neutrophils). *L. major* parasites can delay apoptosis during *in vitro* infected blood neutrophils or *in vivo* infected peritoneal neutrophils (136–138), but exposure of phosphatidylserine, often employed as a surrogate marker of apoptosis, has been reported within a few hours after infection of either bone-marrow derived (135) or dermal neutrophils (139), suggesting parasite-induced induction of apoptosis. After 18 h of infection by *L. braziliensis* peritoneal neutrophils also showed higher levels of PS exposure in the membrane (138). In addition, a few hours after the phagocytosis of *L.a*. neutrophils become annexin V positive, in a process that is driven by NADPH-dependent ROS production (67). It is important to highlight that neutrophil death may be induced by *Leishmania* phagocytosis, as shown by studies employing *Leishmania*-RFP expressing parasites, which allow for comparison of infected versus uninfected cells in the same environment, either *in vitro* or *in vivo*. This is relevant because variations in the infection rate can significantly impact the degree to which the total population will exhibit an infection related phenotype.

The engulfment of infected apoptotic neutrophils or phagocytosis of parasites by other phagocytic cells that are concurrently involved in uptake of apoptotic blebs, a process referred to as efferocytosis, can enhance the chances of parasite survival. In the *Leishmania* literature, this process is commonly referred to as the “Trojan horse” model of infection (140). The neutrophil engulfment by dermal macrophages is mediated by the expression of tyrosinase kinases receptors, such as Axl and MertK, and has been recently shown during *in vivo* infection with *L. major*, where an up-regulation of wound-healing phenotype markers, such as resistin-like molecule alpha (Relmα) and Arg1 was reported (135). *In vitro*, the uptake of *L.m.* and *L.a.* infected apoptotic neutrophils by macrophages enhances transforming growth factor beta (TGF-β) and prostaglandin E_2_ (PGE_2_) production (140, 141). Meanwhile, when dermal DCs acquire *L.m. via* infected neutrophils, an impairment on antigen presentation and T cell priming is observed and is associated with decreased levels of MHC II, CD40 and CD86 expression by DCs (139). Therefore, it appears that *Leishmania* can take advantage of the anti-inflammatory bias that macrophages or DCs acquire after phagocyting apoptotic neutrophils at the site of infection (135, 139). The importance of neutrophil apoptosis in regulating tissue pathology during infection is highlighted in the studies of *L.a.* infection of ROS/NADPH-deficient *gp91^-/-^* mice mentioned above. In these studies, parasite loads are unaffected but NADPH-mediated progression of neutrophils to an apoptotic phenotype is abrogated, resulting in the accumulation of necrotic neutrophils and severe tissue pathology (67).

#### Impact of Neutrophil Deficiency on *Leishmania* Infection

Interestingly, *L.m* can survive but do not appear to replicate within neutrophils during the acute innate phase of the disease (9, 10, 142). The subsequent host protective versus pathogenic roles played by neutrophils during *Leishmania* infection remains somewhat controversial. For instance, depletion of neutrophils in C57BL/6 mice just prior to exposure to the bites of *L. major* infected sand flies resulted in both a reduction in the number of productive transmission events and lower parasite loads (10). Similar observations were reported in neutrophil-deficient C57BL/6 mice infected with *L. mexicana* (143) and, more recently, enhanced neutrophil recruitment mediated by sand fly salivary proteins was also found to facilitate *L.m.* infection (132). On the other hand, depletion of neutrophils prior to a footpad infection in C57BL/6 mice with *L. major* was associated with an enhanced number of parasites in the draining lymph nodes (dLNs), suggesting a protective role for the neutrophils in this context, while the opposite was observed for BALB/c mice (144). In contrast, dermal needle inoculation of neutrophil-depleted BALB/c mice with *L.a.* resulted in increased lesions and parasite loads at the site of infection, while it did not affect the disease outcome in C57BL/6 mice (128, 131). It should be noted that interpretation of mouse studies can be complicated by the use of different *Leishmania* species and the site of infection employed, where intradermal inoculation in the ear is associated with robust neutrophil recruitment and a high proportion of infected cells being neutrophils, similar to sand fly transmission, versus sub-cutaneous inoculation in the footpad that is associated with limited neutrophil recruitment and infection (134, 145). In addition, some studies have employed the draining lymph node to determine parasite load without taking into account the impact of neutrophil depletion on dissemination from the skin or site-specific parasite loads.

#### Neutrophil Antimicrobial Mechanisms

Neutrophils have several antimicrobial mechanisms, such as: phagocytosis, degranulation, ROS production and release of neutrophil extracellular trap (NET), in a process known as NETosis (**Figure 2**) (146), and evidence is still being generated as to the relative importance of each of these mechanisms and the degree to which they can prevent *Leishmania* infection. Specifically, ROS is produced by murine neutrophils after uptake of promastigote or amastigote forms of *L.a. *(66). However, it is unlikely that ROS plays a major role mediating *L.a.* killing as genetic deficiency in the NADPH oxidase gp91 subunit, required for NOX2 ROS production, does not significantly impact parasite load during either the innate or adaptive phases of the disease (65, 67). Neutrophil degranulation during *L.a.* infection has also been reported in both human and murine settings (23, 97). Upon activation by skin extracellular matrix components (MEC), such as fibronectin, during transmigration, neutrophils degranulate releasing MPO, matrix metallopeptidase 9 (MMP9) and neutrophil elastase (NE), all of which can mediate parasite killing by infected macrophages *in vitro* (147). The degranulation of MPO and MMP9 by infected human neutrophils can mediate *L.a.* killing *in vitro via* an LTB4 dependent mechanism (23). In murine settings, increased MPO and NE has been associated with lower *L.a.* numbers in IFNAR^-/-^ mice (97). NETs, primarily containing chromatin and coated by nuclear, cytosolic, and granular proteins as well as microRNAs are released into the extracellular milieu upon stimulation from either naïve or PMA-activated neutrophils (148–150). NETs can be found in cutaneous leishmaniasis biopsies, and their release can be induced by either promastigotes, amastigotes or *L.a.* purified LPG (150, 151). Other species such as *L. infantum*, *L. major* and *L. mexicana* can also induce NETs release by naïve neutrophils (143, 150). Histone dependent NETosis mediated *L.a.* killing within 10 minutes of parasite-neutrophil interactions, in a process that is depended on NE activity. Promastigotes induced higher amounts of NETs released and are also more susceptible to NETosis comparing to amastigotes forms of *L.a* (150–152). Lastly, NETs can impair, *in vitro*, monocyte maturation to a fully differentiated DC phenotype by downregulating IL-4R expression, instead inducing differentiation into macrophages (153). An intriguing open question in the field is how neutrophils “choose” between NETosis or phagocytosis, as currently the understanding is that the two processes are mechanistically mutually exclusive and irreversible. For NET release the enzymes NE and MPO must reach the nucleus to mediate chromatin decondensation, requiring their release from the granules into the cytosol. Meanwhile, during phagocytosis both enzymes are found in the vesicles associated with the endocytic pathway (154). Despite all the effector mechanisms triggered by neutrophils after *L.a.* infection, most parasites survive and are able to initiate the disease. *L.a.* parasites can evade NET mediate killing by LPG, gp63 and 3’nucleotidase/nuclease enzyme expression, which can cleave the NETs, releasing the promastigotes (151, 155). In addition, by expressing peroxidases, *L.a.* amastigotes are highly resistant against ROS production (66, 156).

While little is known about the role of neutrophils during chronic stages of Leishmaniasis, one study has shown that, at least in the case of *L. mexicana*, amastigotes can replicate inside neutrophil phagolysosomes, which expand in volume as described for macrophages (157). Neutrophils were also found to be the predominant infected population at dermal sites of early *L. amazonensis* infection in settings of monocyte deficiency, suggesting they can support a degree of ongoing infection (43). At sites of secondary challenge with *L. major*, where the majority of parasites are eliminated by IFN-γ activated inflammatory monocytes, a significant proportion of the parasites that are remaining, are found in neutrophils. Infection at secondary sites under conditions of monocyte deficiency is also associated with infection of neutrophils, suggesting that even in settings of robust pre-existing Th1 immunity, neutrophils remain a safe haven for infection (142). While these observations suggest neutrophils can maintain infection and even proliferation, studies implicating the mononuclear infiltrate as the host population that maintains parasite replication during the highly regulated late stages of the disease suggest neutrophils play a minor role in the ongoing maintenance of the parasite load under normal conditions (43, 134, 142, 157). In addition to directly providing a permissive host cell for parasites, neutrophils can also modulate the immune response by releasing several chemokines that promote monocyte, DCs, and T cells recruitment to sites of inflammation (158, 159).

Ultimately, any protective mechanism that does not achieve sterilizing immunity is, in fact, non-protective if infection can be initiated by a single parasite, as previously shown (9), and progress to the same peak parasite load and lesion size, albeit with different kinetics. Under this premise, the key biological events to take place in neutrophils, despite all the associated effector mechanisms, would be the differentiation from promastigotes to amastigotes, the stage adapted to survive within phagosomes, in addition to the modulation of cytokine/chemokine production and by potentially facilitating the silent entry of amastigotes into other phagocytic cells.

## Resident and Monocyte-Derived Dendritic Cells

**Figure 3:**
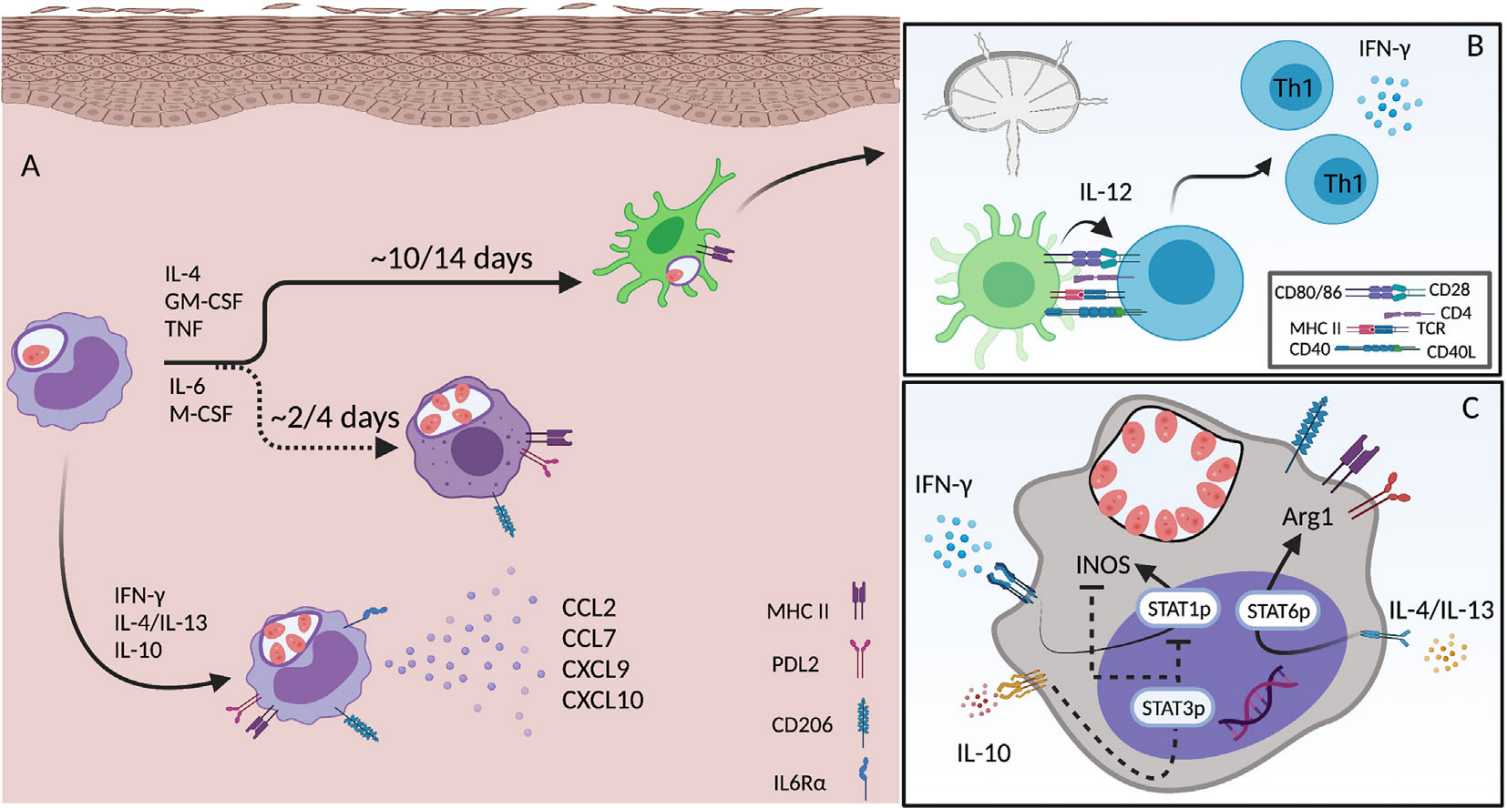
Monocyte differentiation and activation during *L.a.* infection. **(A)** Infected inflammatory monocytes acutely recruited to the site of infection become an important source of chemokines (CCL2, CCL7, CXCL9 and CXCL10) when activated by CD4 T cell derived IFN-γ. Infection triggers IL-6rα expression and signaling, which is important for the differentiation towards a macrophage phenotype. Mon-Macs are seen 2-4 days after monocyte recruitment to the site of infection. When activated by Th1 and Th2 cytokines both monocytes and mon-Macs upregulate the expression of MHC II, PDL2 and CD206. Inflammatory monocytes can also differentiate into DCs, a longer process that takes approximately 7-14 days. mon-DCs upregulate MHCII, but not PDL2 or CD206, and are critical for the priming and induction of Th1 immunity **(B)**. The phenotype of both monocytes and mon-Macs exposed to different cytokines produced by different CD4 T cell subsets, leading to, in a single cell, activation of STAT1, STAT3 and STAT6, allowing the co-expression of both iNOS and Arg1 **(C)**. Created with BioRender.com.

Dendritic cells were firstly described in the late 1970s (160) and are often referred to as the cells that “bridge” the innate and adaptive immune responses. DCs are a heterogenous population and different subsets have been described according primarily to their ontogeny and function. In the skin dermis conventional DCs (cDC1 and cDC2), minor populations of CD11b^-^ DCs, such as plasmacytoid DCs (pDCs), and monocyte-derived DCs (mon-DCs) have been described (161). As professional antigen presenting cells (APCs), DCs can migrate from peripherical tissues to present antigen and activate naïve CD4 T cells in secondary lymphoid organs such as dLNs (**Figure 3**). Amongst the APCs active during infection with *Leishmania*, DCs represent the major source of IL-12 production and are critical for the development of Th1 immunity (162). After pathogen phagocytosis and during the migratory process, DCs undergo a process of further maturation, upregulating the expression of co-stimulatory molecules (CD86, CD80, CD40) and MHC II, which are essential for T cell priming (**Figure 3B**). The duration of this process presents an opportunity for pathogens to interfere with DC maturation and impair subsequent T cell priming (163). This is especially true for monocyte derived DCs that originate as CD11c^-^MHCII^-^ cells in the blood and are permissive to *L.a.* replication (43). The specific role played by each DC subset during *Leishmania* infection is still not completely understood. Important questions such as if different DC subsets play a different role during (a) early versus late phases of the disease, (b) at the site of infection versus dLNs and (c) if and how they interact with each other remain to be elucidated.

### cDC1, cDC2, and pDCs

cDC1s are a minor population in the skin dermis of both humans and mice while cDC2s are the most abundant (161). cDC1s are usually associated with the cross-presentation of exogenous antigens and priming of CD8^+^ T cells and with the development of Th1 CD4 immunity. The role of cDC1s during *Leishmania* infection has been assessed using Baft3 (Basic leucine zipper transcriptional factor ATF-like 3) deficient mice, as cDC1 development is reliant on the expression of this transcriptional factor. During infection with *L. major*, Baft3^-/-^ mice presented with a deficit in Th1 immunity and enhanced frequencies of Foxp3^+^ T regulatory cells in the skin that was associated with non-healing lesions and higher parasite loads versus Wt mice (164, 165). This occurred despite the fact that the priming of CD4^+^ or CD8^+^ T-cell responses was driven primarily by Baft3-independent DCs. Because Baft3 dependent DCs represent an important source of IL-12 in the skin, they likely impact Th1 CD4^+^ cells and CD8^+^ T cells activation at the site of infection despite their low numbers (164). We can also speculate that, as Baft3 dependent DCs represent a source of the chemokines CXCL9, CXCL10 and CXCL11 during bacterial infection, they may also participate in the recruitment and retention of CXCR3^+^ Th1 effector cells to the site of infection (166). Thus, it is not clear if the reduced numbers of Th1 cells in the dermis after *L.m.* infection is due to a deficit on T cell recruitment or/and in-situ activation mediated by IL-12 produced by cDC1s. Baft3 dependent DCs also play a protective role during *L. infantum* infection, as higher numbers of parasites were observed in the liver, but not spleen or bone-marrow, during the late stages of disease (7 weeks post-infection) in Baft3^-/-^ versus Wt mice (167).

A few caveats associated with the use of Baft3 deficient mice to study the role of cDC1s or cross-presentation should be taken into account. cDC1 development may be restored in certain tissues under inflammatory conditions, probably due to the redundant role played by other BAFT proteins. In addition, while cDC1s are absent in the skin and spleen of Baft3^-/-^ mice, these APCs or a very similar population are still present in lymph nodes (161, 168). In addition, other APCs, such as monocytes-derived DCs can cross-prime CD8^+^ T cells. In this case, monocytes rely on the expression of interferon regulatory factor 4 (IRF4), but not Baft3, to differentiate into DCs that mediate this process (169). It is important to mention that Baft3 deficient mice also show an impairment in monocyte maturation towards a DC phenotype. At early stages after *L.m.* infection (2-3 weeks), most Baft3 deficient monocytes remained Ly6C^+^MHCII^-^, indicating additional roles for Batf3 in different dermal APCs that could have significant secondary impacts during *Leishmania* infection (164). In regards to cross-presentation, it is known that *L. major* can impair the canonical endoplasmic reticulum transporter associated with antigen processing (TAP)/proteasome-dependent pathway, delaying CD8^+^ T cell priming (170). It is still not well described how peptides are processed for *Leishmania* cross-presentation but gp63 from *L.m* can cleave the SNARE VAMP8, present on late endosomes, preventing NADPH oxidase complex assembly, which impacts the phagosome pH and proteolytic activity, inhibiting cross-presentation (32). The role of cDC1s or cross-presentation specifically during *L.a.* infection is unknown, but macrophages can prime CD8^+^ T cells by presenting endogenous *Leishmania* antigen *via* the classical proteasome-dependent pathway (171).

cDC2s are usually associated with the development of a Th2 immune response, which requires IL-4 and alarmin (thymic stromal lymphopoietin (TSLP), IL-25 and IL-33) signaling (161). While cDC1s migrate towards the T cell zone of the lymph nodes, cDC2s localize at the subcapsular sinus space (172). Thus, in addition to expressing CCR7 to migrate into dLNs, CD301b^+^ cDC2 cells rely on CCR8 expression, during allergic immune response, to reach the LN parenchyma to trigger Th2 immunity (173). Thus, the anatomical location of different DCs subsets at dLNs play a significant role to determine the phenotype of adaptive immunity (174). To date, the exact role played by cDC2s on the establishment of the Th2 response during *Leishmania*, including *L.a.*, infection is not clear.

Plasmacytoid DCs (pDCs) are found in the circulation in steady state but can be recruited to the skin during inflammation. These APCs are CD11b^-^ CD11c^+^and represent an important source of type I IFNs (IFN-α/IFN-β), thus are critical for protective immunity during viral infections (161, 175). pDCS, when infected *in vitro* with *L. infantum*, can also induce type I IFNs, in a TLR9 dependent manner, in addition to IL-12 (176). During *L. major* infection pDCs can be found at skin dermis and dLNs, can be infected by the parasite and do not seem to produce significant amount of NO, which suggest these cells do not play a role in parasite killing (177). Compared to CD11b^+^ DCs, pDCs produce higher levels of IFN-α/β and lower levels of IL-12 in response to *L. major* infection (178). Type I IFNs have been associated with early iNOS expression at the site of infection during *L. major* infection (179) but a lack of type I IFN signaling does not impact the self-healing phenotype after *L. major* infection of C57BL/6 mice suggesting this function of pDCs might be dispensable (178), but more studies are still required.

### Monocyte-Derived Dendritic Cells

While cDC1s might have an important role on eliciting Th1 effector cell activation in the skin, monocyte derived DCs seem to play the major role in priming the Th1 immune response in dLNs and mediate parasite elimination. Monocytes are recruited to the dermis after infection, differentiate into mon-DCs, and after migrating into dLNs prime Th1 immunity by producing IL-12 (180). In different experimental models, CCR2 deficient mice have been associated with a profound Th1 deficit and skewing towards Th2 immunity. Monocytes rely on CCR2 expression to leave the bone-marrow and reach the circulation, thus without this chemokine receptor monocytes are not available to enter peripherical tissues (181). The shift towards a Th2 immune response in CCR2^-/-^ mice has been reported for infection by *L. major* (182) and *L.a* (43). During *L. mexicana* infection, the observed decrease in monocyte recruitment, versus *L. major* infected mice, may also be responsible for decreased Th1 priming (183). In addition, mon-DCs can express iNOS and represent an important cell that mediates parasite killing during *L.m.* infection (184). In contrast to *Baft3^-/-^* mice that have non-healing but stable lesions, monocyte-deficient *ccr2^-/-^* mice have highly significant deficiencies in iNOS^+^ cells and succumb to progressive disease at the late stages of infection in otherwise resistant C57BL/6 mice (164, 182, 184).

Mon-DCs are likely infected with amastigotes, rather than promastigotes. *In-vitro* infection of DCs by amastigotes has an important impact on activation and is associated with an overall anti-inflammatory response, associated with poor inflammasome activation, IL-12 production and CD4 T cell proliferation. How *L.a.* amastigotes compromise DC activation is not fully understood, but it does affect several important transcription factors signaling pathways, including: (a) reduces expression of IRF1, IRF8 and STAT1; (b) degrades STAT2 by proteases, (c) inhibits the NF-kB pathway by enhancing optineurin (OPTN) expression (118, 185). A caveat to the interpretation of these studies is that mon-DCs are likely to be infected when they are immature monocytes (43, 142, 180, 184), and therefore the relevant impact of infection may be the modulation of maturation towards a DC phenotype rather than the down-regulation of a cell that is already mature, such as is the case with bone-marrow derived DCs typically employed for these types of *in-vitro* experiments. These two processes are biologically distinct.

### Parasite Acquisition by DCs

*L.a.* (both promastigotes and amastigotes) can infect and proliferate in both murine and human differentiated DCs through different receptors such as FcγR, complement, and proteoglycan receptors (186–189). Monocyte differentiation into DC is impacted by *L.a.* infection *in vitro*, as lower levels of CD80 and CD1a expression and IL-6 production were reported. However, the infection did not impact the expression of MHC II and increased CD86 levels, showing that this modulation does not occur equally across all maturation markers (189). How *L.a.* impacts DC activation varies between different studies because of differences on parasite stage, opsonization or not, genetic background and maturation status at the time of infection. In summary, parasite opsonization, either amastigotes or promastigotes, by antibodies, but not complement, plays a major role on DC activation. For DCs generated from BALB/c mice ab-opsonization of metacyclic promastigotes or amastigotes is associated with higher expression of CD40, CD86, CD54 and OX40L. In addition, ab-opsonization not only enhanced MHC II expression but it also accelerated its distribution on the cell surface, impacting antigen presentation (186). On the other hand, infection by non-opsonized metacyclics did not alter DC activation (186). Meanwhile, DCs infected with non-opsonized stationary promastigotes were able to weakly up-regulated MHC II, CD40, CD80 and CD86 expression (185, 187, 190). In DCs derived from C57BL/6 mice the use of non-opsonized metacyclic inhibited the expression of molecules such as MHC II, CD40, CD86 and IL-12p70. Particularly, the expression of CD40 is inhibited by the enhanced expression of DCs ectonucleotidases (CD39 and CD73) induced by *L.a.* infection, which is IL-4/IL-10 independent but ERK1/2 pathway dependent (191–194). These discrepant results reinforce the fact that the use of purified metacyclics has a big impact on infection, similar to opsonization. By inhibiting CD40 expression on DCs, *L.a.* might impair the development of a protective adaptive immune response as CD40 expression is relevant for IL-12 production and subsequent Th1 priming (195), including during *L.a.* infection (191, 196). In fact, *L.a.* parasites do impair both CD40 and IL-12 production on APCs to a higher degree then what is observed for self-healing parasite strains of *L. major* and *L. braziliensis* (190, 191). In summary, the infection of DCs by either metacyclic promastigotes or amastigotes have relevant impact on promoting anti-inflammatory response that may delay Th1 priming.

Other important questions related to DCs and still to be elucidated are: 1) if cDC1s represent an important source of IL-12 during early stages of the disease, before the recruitment of monocytes to the site of infection; 2) if continuous local activation of cDC1s, after the priming of Th1 immunity by mon-DCs in dLNs is required for ongoing protection; and 3) why the dermal resident cDC1s, which are already at the site of infection are not required for Th1 priming. This is a relevant point because cDC1s have higher migratory capacity and can induce stronger T cell activation, for both CD4^+^ and CD8^+^ T cells *in vitro* (197), compared to mon-DCs. However, as cDC1s might become immobile after, at least, the first hours post-infection, due to initial infection and inflammation at the dermis (198), we can speculate that, in addition to the fact that these are rare cells at the dermis, this immobilization might impact their priming capacity.

## Monocytes and Macrophages

### Macrophage Ontogeny Matters

Embryonic-derived macrophages, Monocytes and Monocyte derived Macrophages (mon-Mac), are mononuclear phagocytic cells that play an essential role during homeostasis and inflammation in different organs, including a critical role mediating killing of intracellular pathogens and promoting wound healing [reviewed in (199, 200)]. During *Leishmania* infection these cells can play paradoxical roles, providing both a niche for parasite survival and replication or mediate parasite killing depending upon the activation state of the tissue environment they occupy (43, 142, 184, 201–203). It is important to highlight that for this review we are considering the current literature for macrophage ontogeny in the context of *Leishmania* infection in the skin. The reason why macrophage ontogeny matters is that in recent years, it has become clear that the ontogeny of macrophages has an impact on macrophage function, including differences on phagocytosis, cluster interaction with other immune cells and production of cytokines and chemokines. Differences on epigenetic and transcriptomic networks may explain these differences, but this is a field currently under intense investigation (197, 204, 205). It is well established that different tissues contain a heterogenous resident macrophage population, based on their origin, which can vary depending upon life history, including age and experience with inflammation. Resident tissue macrophages (RTMs) can originate from embryonic hematopoiesis and are self-maintained by replication or originate from the recruitment and differentiation of circulating CCR2^hi^Ly6C^hi^ monocytes after birth (197, 199). In vascularized tissue-barrier organs such the skin dermis, the replacement of embryonic derived macrophages by monocyte-derived cells is observed after birth and occurs more quickly compared to tissues such as the epidermis, brain and liver. After 36 months of age, around 80% of RTMs are monocyte-derived in the skin dermis (206, 207). It is relevant to mention that during cutaneous leishmaniasis two waves of monocyte recruitment can be observed. The first wave starts around 48 h post-infection and last up to around 72 h. Then, another monocyte wave is seen during adaptive immunity, starting around day 10 post-infection, and will persist for as long as it takes for parasite clearance (43, 139). The impact of this is that recently recruited immature monocytes, monocytes undergoing different stages of maturation toward a macrophage or DC phenotype, and fully differentiated monocyte-derived macrophages and DCs will co-exist at the site of infection. There are two main challenges when trying to define the role played by embryonic-derived macrophages, monocytes and mon-Macs: 1) studies relying on markers such as MHC II, F4/80 and CD11c to distinguish between these cells, and sometimes DCs, which we now know are not definitive, in addition to the fact that several studies do not take into account ontogeny when referring to macrophages (197). The most current markers to define innate cells at the skin can be seen in **Figure 1**, 2) much of what we know about macrophage function came from BMDMs, where monocytes are first fully differentiate towards a macrophage phenotype *in vitro*, and then infected by the parasite. The caveat here is that in most models of *Leishmania* infection, including *L.a.*, recruited monocytes appear to be the cell type associated with ongoing parasite replication, though further work is required (43, 142, 208–211). Lastly, in addition to mon-Macs, a recently defined relevant macrophage population in a context of cutaneous leishmaniasis are the self-renewing embryonic-derived RTMs, which are maintained in an IL-4 dependent manner, which impacts the ability of these cells to get activated in a context of Th1 immunity (212). These cells in the dermis are characterized by high levels of MR expression and represent one of the first host cells for the *L. major-RYN* strain after sand fly transmission, which, at least in C57BL/6 mice, has a non-healing phenotype, and the *L. major Seidman* strain after needle inoculation, which exhibits a non-healing phenotype in both C57BL/6 mice and the patient from which it was derived (135, 201).

Finally, to best understand whether these cells provide a safe or a hostile environment for *Leishmania* parasites we cannot simply rely only on analyzing their activation status. Rather, we propose that to understand the big picture of the role of mononuclear phagocytic cells a balance between three equally important factors must be taken in consideration: 1) activation, 2) recruitment, and 3) spatial organization in the tissue.

### Monocyte/Macrophage Activation

Monocytes and Macrophages show high plasticity and heterogeneity in regard to their activation phenotype, which is determined by environmental cues. Depending on the cytokines, PAMPs and damage associated molecular patterns (DAMPs) in their environment, they can acquire different functions driven by both innate and adaptive immune responses. When exposed to cytokines such as IFN-γ and TNF these cells acquire a pro-inflammatory phenotype (or “M1-like”) and are able to produce high levels of iNOS, ROS, and cytokine and chemokine production such as TNF, CXCL9 and CXCL10. When activated by IL-4 and/or IL-13 they are associated with a wound-healing function (or “M2-like”). In this case these cells express molecules such as Relmα, Ym1, CCL24 and Arg1. If activated by immunocomplex or PGE_2_ these cells can play a suppressive function by producing high levels of anti-inflammatory cytokines such as IL-10 (200, 213, 214). Therefore, in the context of Th1 immunity these mononuclear cells, activated by IFN-γ to produce NO can control *Leishmania* growth (70, 78, 215, 216). Higher iNOS expression from skin biopsies from patients with American cutaneous disease has been associated with LCL and smaller number of parasites comparing to patients with DCL and higher parasite burden in the lesions (217). On the other hand, the expression of host cell Arg1 in highly Th2 polarized immune responses, such as infection of BALB/c mice, has been associated with parasite replication and susceptibility (59, 218, 219). Importantly, in a context of cutaneous leishmaniasis embryonic-derived macrophages, due to close cluster interaction with eosinophils, and discussed in more detail below, show a wound-healing phenotype despite the overall polarized Th1 immune response in the dermis (212).

In the context of *Leishmania* infection, monocytes, and both embryonic and monocyte-derived macrophages can also be suppressed by IL-10, which compromises the induction of effector mechanism, such as TNF, ROS and NO production, facilitating parasite growth or persistence (43, 201, 220–222). Most of the anti-inflammatory functions associated with IL-10 is associated with STAT3 signaling. STAT3 can induce the production of proteins that suppress the expression of several pro-inflammatory genes such as the suppressor of cytokine signaling 3 (SOCS3) and can inhibit NF-kB translocation by inducing IL-10 production (223), In addition, STAT3 can cross-regulate STAT1 signaling, the key transcription factor associated with IFN-γ activation. Depending on the experimental model, STAT3 can interfere with STAT1 tyrosine phosphorylation or can promote STAT1 sequestration, by binding to STAT1 forming STAT1-STAT3 heterodimers, which inhibits the DNA binding of STAT1 homodimers (224). The production of IL-10 by phagocytic cells during *Leishmania* infection is associated with ab-opsonized amastigotes, through binding to FcγR, inducing ERK1/2 activation, leading to IL-10 production (221, 225, 226). In addition, both Tregs (CD4^+^CD25^+^FoxP3^+^) as well as Th1 (CD4^+^CD25^-^FoxP3^-^T-bet^+^IFN-γ^+^) cells have been described as important sources of IL-10 during *L. major* and *L. donovani* infections (227, 228). Importantly, the lack of IL-10, however, is not enough to revert the susceptibility phenotype seen in C57BL/6 mice infected with *L.a.*, despite the stronger Th1 immune response (43, 221, 229).

During *in vivo* infections by *L.a.* parasites, a mix of different CD4 T cell responses is observed (Th1, Th2), in addition to the presence of other cell types such as NK cells and ILC2, which can produce IFN-γ or IL-4 for instance (212, 230, 231). Thus, in addition to mononuclear phagocytic cells presenting highly differentiated M1-like or M2-like phenotypes in the skin lesions, the same cells, often simultaneously, express iNOS and Arg1, indicating that they are activated by diverse stimuli (**Figure 3C**) (43, 232). In fact, during *L.a.* infection most iNOS^+^ cells are also Arg1^+^ cells, even in a context of a Th1 prone environment (43), which could potentially prevent, in a single cell-manner, robust NO production by these cells, as previously described for *L.m*. infection (232). Our group also showed that infection of STAT6 deficient mice on the C57BL/6 background was associated with an almost complete abrogation of Arg1 expression by phagocytic cells, but this did not restrict parasite growth. Rather, by upregulating genes associated with L-arginine uptake and its own *arginase* versus WT infected mice, *L.a.* do not rely on host arginase to acquire polyamines, which is probably also true for other parasite species (43). For instance, in contrast to BALB/c infected mice, *L. major* growth in C57BL/6 mice also occurs independently of host Arg1 expression (233).

#### Adaptive-Innate Cross Talk and Its Impact on Infection

**Figure 4:**
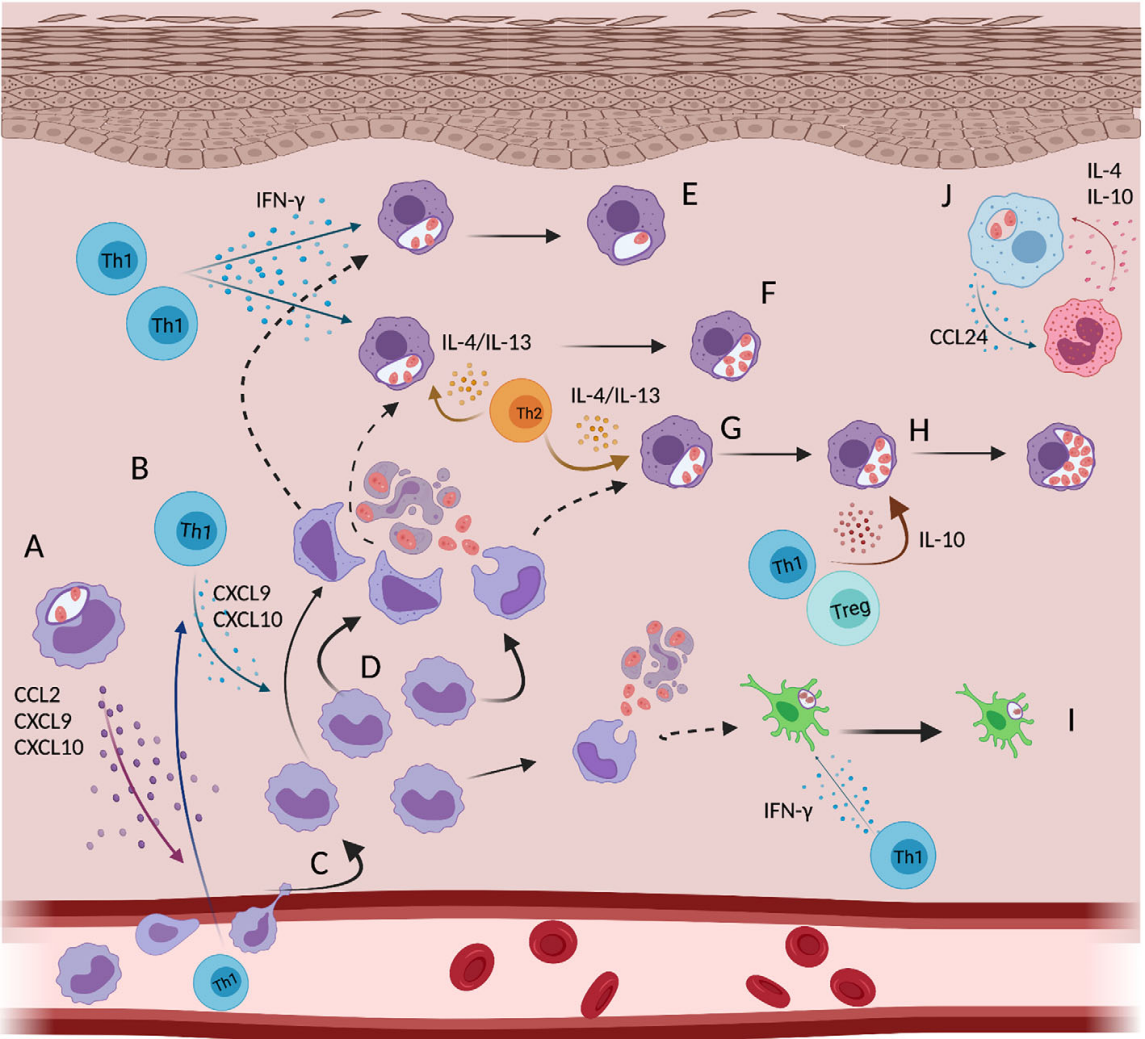
Dynamic of monocyte and monocyte derived cells during an ongoing adaptive immune response against *L.a.* infection: recruitment, activation and cell clusters. Infected monocytes **(A)** and CXCR3^+^ Th1 cells **(B)** produce CXCL9 and CXCL10 facilitating continuous recruitment of permissive immature inflammatory monocytes **(C)** that provides a niche for ongoing infection **(D)** and possibly favors the formation of monocyte-Th1 cell clusters. After infection, mon-Macs mediate some parasite killing when activated by IFN-γ **(E)**; but when activated simultaneously by IFN-γ and Th2 cytokines **(F)** or by Th2 cytokines alone **(G)** support parasite replication. Infected monocytes can also be suppressed by IL-10, while receiving signals from IFN-γ and/or Th2 cytokines, which also favors parasite replication **(H)**. Mature mon-DCs are more efficient at restricting parasite growth versus mon-Macs but this developmental program takes 7-14 days **(I)**. Embryonic derived resident macrophages due to CCL24 production, cluster with eosinophils, which are an important source of IL-4 and IL-10 **(J)**. Created with BioRender.com.

The idea of a weaker Th1 immune response during *L.a.* infection compared to *L. major* infection has been implicated as a cause of susceptibility (230, 234). However, this weaker Th1 immunity appears to be dose and/or route of infection dependent, as, following intradermal infection with lower doses, this phenotype is not readily apparent (43, 235). In addition, we and others have shown that the enhancement of Th1 immunity does not translate into a protective phenotype, despite the strong induction of iNOS expression by phagocytic cells (43, 79, 229, 236). There is no doubt that IFN-γ do mediate parasite killing during *L.a.* infection, however, the exponential parasite growth observed during early stages of the disease is not impacted by the absence of IFN-γ (43, 44, 236). We propose that, rather than eliciting a weak Th1 response or compromising the induction of this response, *L.a.* parasites have evolved to actually take advantage of some aspects of the otherwise protective type I immune response to establish a chronic and progressive disease (**Figure 4**). IFN-γ, the key cytokine produced by Th1 cells, modulates immunity through different ways, such as inducing effector mechanisms (NO/ROS production) in innate cells, but also by influencing immunometabolism, leukocyte trafficking, apoptosis and cell proliferation, to name a few (237). Two properties of IFN-γ function can favor *L.a.* growth: 1) it’s influence on cell metabolism and 2) leukocyte trafficking to skin lesions. When BMDMs are pre-activated with low doses of IFN-γ, and then infected with *L.a.* amastigotes *in vitro*, an enhancement in parasite replication, rather than their killing is observed. In this case, IFN-γ mediate CAT-2B up-regulation by BMDMs, increasing, therefore, the availability of this amino acid for *Leishmania* metabolism (60, 61). In addition, IFN-γ mediates the production of chemokines such as CCL2, CCL5, CXCL9 and CXCL-10 (44), significantly increasing leukocyte trafficking to sites of inflammation. In the context of *L.a.* infection, the influence of IFN-γ on monocyte recruitment has a large impact by providing host cells for parasite growth, as discussed below.

### Monocyte Recruitment

The observation that equivalent numbers of *L.a.* infected cells are seen in lesions of STAT6^-/-^ mice compared to Wt mice, despite strong Th1 immunity, greater frequencies of iNOS^+^ phagocytic cells, and the absence of Arg1 expression is explained by an enhanced monocyte infiltration and IL-10 production. The opposite is found in *ifnγ*-deficient settings, where a lack of a Th1 response during the early phases of infection leads to a Th2 polarized environment but no enhancement of parasite numbers due to a lack of Th1-mediated monocyte recruitment (43). These data show how important the recruitment of permissive monocytes to the site of infection can be, which has been shown in different contexts for other *Leishmania* parasites as well (208, 211). In fact, emergency hematopoiesis, a process that facilitates the expansion of hematopoietic stem cells (HSCs) and myeloid progenitor cells (MPCs) is stimulated by systemic infection with *L. donovani* and is largely associated with the expansion of Ly6C^hi^ monocytes rather than neutrophils, thereby providing a robust and ongoing supply of infiltrating monocytes that are permissive to infection (209). When in the tissue, monocytes differentiate into macrophages (mon-Macs) or DCs (mon-DCs), a process that takes time (**Figure 3A**). The different environmental cues, in different experimental models, associated with monocyte differentiation towards either mon-Macs or mon-DCs are still not completely understood. IL-4, TNF and GM-CSF has been associated with a mon-DC phenotype (238, 239) while IL-6 and M-CSF with a mon-Mac phenotype (240). If present during early stages of monocyte differentiation, IFN-γ, by modulating IL-6 expression and M-CSF expression and internalization, can shift human monocyte differentiation, *in vitro*, from a DC to a macrophage phenotype, even in the presence of IL-4 and GM-CSF (241). In addition, germ-free mice, under steady state, present lower numbers of mon-DCs in the dermis, but the same number of mon-Macs comparing to specific pathogen free mice, showing that the microbiota also influences monocyte fate in the skin (197).

Employing a tamoxifen inducible CCR2-Cre lineage tracing strategy we found that maturation from a circulating CCR2^+^Ly6C^+^ phenotype to a CCR2^-^Ly6C^-^CD11c^+^ DC phenotype requires approximately 2 weeks, during which the majority of maturing monocytes provided a safe niche for parasite replication, despite the Th1 immune response, likely as a result of *L.a.*-infection induced manipulation of cell activation by enhancing IL4R, MR and PDL2 expression on infected versus uninfected monocytes from the same dermal site of infection (43). While Mon-DCs represent the cell phenotype that is most efficient at restricting *L.a.* growth and adopts an MHC^+^PDL2^-^ phenotype, even these cells can be found to harbor viable parasites, which has been shown for other pathogens (43, 184, 242). Importantly, permissive monocyte recruitment is mediate by IFN-γ produced by Th1 cells, as depletion of CD4^+^ T cells abrogated virtually all IFN-γ mRNA from the lesion site 21 days post-infection. Lastly, the monocytes and monocyte derived cells, recruited after the development of Th1 immunity, which coincide with parasite exponential growth, represent the host cells that harbor higher numbers of amastigotes, in contrast with the cells recruited during the innate phase of the disease and that are further along in their maturation (43). Altogether, these data suggest that *L.a*. parasites have evolved through evolution to subvert this property of Th1 immunity to facilitate infection. This represents one of the strongest examples of the paradox of a phagosomal lifestyle, where *L.a.* parasites evolved to depend upon monocyte recruitment mediated by IFN- γ to provide a permissive niche for replication.

### Tissue Spatial Organization

During infection with the non-healing Seidman or Ryan strains of *L. major*, embryonic derived RTMs, *via* close interactions with IL-4 producing eosinophils, maintain an M2-like, wound healing, activation state, despite highly polarized Th1 immunity at the site of infection, providing a perfect niche for replication (135, 212). In addition, monocytes and monocytes derived cells represent the majority of phagocytic cells responding to Th1/Th2 cytokines during infection with either the *L.a*. or *L.m*. (healing Fn) strain (43, 232). In CCR2 deficient mice, a huge deficit in the number of phagocytic cells with either a iNOS+ or Arg1+ phenotype strongly support the idea that monocytes and monocyte-derived cells are the cells either directly interacting with T cells or that are in close enough proximity to become activated by the T cell cytokine gradient (43). Specifically, Th1 CD4 T effector cells can mediate protection by interacting with only a fraction of infected cells, as a gradient of IFN-γ can reach and activate phagocytes that are not directly interacting with T cells to promote pathogen control. Furthermore, to reach an effective killing activity a certain threshold of phagocytic cells expressing iNOS might be necessary, where due to the diffusion capacity of NO through membranes, non-activated host cells are also able to kill the parasite (75, 243). In addition, dermal *L.a.* infected monocytes (43), as well as Th1 effectors cells, produce and are chemoattracted by the chemokines CXCL9 and CXCL10 (244, 245), which might favor cluster formation between these cell types (**Figure 4**). Lastly, the migratory capacity of Th1 and Th2 cells in the skin is different. Th2 cells, by expressing higher levels of the integrin αVβ3, can undertaking a broader “scanning” phenotype in the tissue and interact with more cells, compared to Th1 cells, which are more dependent on chemokine gradients to migrate (246).

We can speculate that ontogeny plays a role in the formation of phagocyte-T cell clusters, as embryonic derived RTMs are less mobile, while both monocytes and T cells migrate to the site of infection *via* the bloodstream, which may favor cluster formation between these cells. Clusters of embryonic derived RTMs associated with local skin nerves and clusters between T cells and monocytes during skin contact hypersensitivity has also been reported showing that the tissue spatial organization influences physiological and immunological events (247, 248). These relatively new data strongly support the idea that cell clusters among different cell subsets at the site of infection plays a major role on the outcome of disease, creating an opportunity for specific niches that facilitate pathogen growth or killing.

## Concluding Remarks

In a context of evolutionary host-parasite interactions the host must evolve and adapt to virulence factors associated with the pathogens, while the parasites have to evolve and adapt to the host-immune response. Phagosomal pathogens, such as *Leishmania*, have adapted to survive and replicate inside the innate cells that are recruited to eliminate them, representing a challenge for effective intervention. Innate effector mechanisms, for reasons discussed in this review, are not enough to eliminate *Leishmania* parasites and that creates an opportunity for the parasite to establish a niche for replication before the induction of Th1 adaptive immunity. During infection with the self-healing *L. major* strain, the development of Th1 immunity is enough to induce protection. However, in the context of *L. amazonensis* infection, Th1-dependent monocyte recruitment provides an opportunity for the parasite to replicate inside recently recruited immature monocytes and, while IFN-γ activation does ultimately provide some parasite killing, these infected cells are also responding to other environmental cues that are not associated with parasite elimination (**Figure 4**). The use of reporter parasites *in-vivo* has been instrumental in revealing the characteristics of infected monocytes and, while these cells eventually adopt characteristics of macrophages or DCs, to a certain degree they represent unique populations of monocyte-derived phagocytes that are more defined by their infection status rather than a classical macrophage or DC phenotype. Mechanistically reaching a subtle balance between monocyte recruitment by modulating the Th1/Th2 immune response to allow parasite killing while not providing a permissive host cell reservoir might represent one of the current big challenges in the field that could potentially lead to a vaccine or improvement of therapy.

## Author Contributions

MBC and NCP wrote the review. MBC made the figures and NCP did the revision of the figures. All authors contributed to the article and approved the submitted version.

## Funding

This work was supported by Canadian Institutes of Health Research grant MOP-142302 and Canadian Foundation for Innovation grant RCP-16-027-SEG to NCP. A Snyder Institute for Chronic Diseases Beverley Phillips Rising Star Fellowship and Cumming School of Medicine Post-Doctoral Fellowship supported MBC. MBC received professional development training and travel funding from The University of Calgary Host-Parasite Interactions Program.

## Conflict of Interest

The authors declare that the research was conducted in the absence of any commercial or financial relationships that could be construed as a potential conflict of interest.

## Publisher’s Note

All claims expressed in this article are solely those of the authors and do not necessarily represent those of their affiliated organizations, or those of the publisher, the editors and the reviewers. Any product that may be evaluated in this article, or claim that may be made by its manufacturer, is not guaranteed or endorsed by the publisher.
